# Sexually Dimorphic Effects of Neuromodulatory Drugs on Normal and Stress-Induced Social Interaction in Rats

**DOI:** 10.3390/brainsci13101378

**Published:** 2023-09-27

**Authors:** Sara Ishaq, Touqeer Ahmed

**Affiliations:** Neurobiology Laboratory, Department of Healthcare Biotechnology, Atta-ur-Rahman School of Applied Biosciences, National University of Sciences and Technology, Islamabad 44000, Pakistan; sarahishaq1622@gmail.com

**Keywords:** social behavior, antipsychotic, antidepressant, acetylcholinesterase inhibitor, stress

## Abstract

Social behavior is a complex term which involves different interactions between various individuals of a community. It is controlled by different neurotransmitter systems in a sexually dimorphic way. Certain environmental factors, like stress, cause various neurological disorders with associated social abnormalities in a sexually dimorphic way. Multiple drugs are used in clinical settings to treat behavioral disorders. However, the sexually dimorphic effects of these drugs, particularly on social behavior, still need to be studied. The present study was designed to investigate the sex-dependent effects of Risperidone, Donepezil, and Paroxetine in 8–12 weeks old male and female rats under normal and stressed conditions. There were four male and four female groups, i.e., control group (no drug treatment), Risperidone (3 mg/kg/day) treated group, Donepezil (5 mg/kg/day) treated group, and Paroxetine (10 mg/kg/day) treated group. Each group received its respective drug during phase 1 for 21 days, followed by a 10-day break with no drug treatment. After the break, same groups received the same drugs along with tilt-cage stress for an additional 21 days during phase 2. A social preference and novelty test was performed at the end of both phases (1 and 2). During phase 1, Risperidone treatment caused impaired social behavior and reduced locomotion in the male group only, compared to its control group. Donepezil treatment caused a reduction in social interaction, while Paroxetine treatment caused increased social interaction and locomotion in a sex-dependent manner. During phase 2, social novelty was affected in both male and female stress groups. Treatment with drugs along with stress showed differential sex-dependent effects. The study showed a predominant effect of Risperidone on males while there were differential effects of Donepezil and Paroxetine on both sexes. This study has paved the way for the development of more targeted and effective neuromodulatory drugs for use against various psychiatric and social deficits.

## 1. Introduction

Social behavior is a complex term, usually defined as interaction between two members of the same species [1]. An appropriate display of social behavior is crucial for an organism’s survival, because it helps one to form community relationships, gain food, achieve reproductive success, and avoid predation [2]. Social behavior is reported to be controlled mainly by the prefrontal cortex (PFC) with its massive reciprocal neuronal connections forming a top-down modulatory system [3,4,5,6]. This neuronal network forms diverse connections with other brain regions, i.e., the amygdala for emotional control, hypothalamus for stress processing, hippocampus for memory formation, nucleus accumbens (NAc) for social reward, and some other cortical regions for sensory and motor input and output [6,7]. These brain regions control social behavior in a sexually dimorphic way via differential activities of various neurotransmitter systems [8,9,10].

The dopaminergic neurotransmitter receptors display a relatively decreased activity in the frontal cortex and amygdala in males [11] and in the nigrostriatal pathway in females [12]. Similarly, estrogen in females is reported to influence adrenergic receptor expression, causing increased norepinephrine levels compared to males, particularly in the locus coeruleus (LC) [11,13,14,15]. Serotonergic receptors also show differential expression, with higher levels of 5HT_2_A receptors in the hippocampi of males and of 5HT_1_A in the hippocampi of females [16]. Similarly, memory retrieval in males and females is based on sex-specific variations in the cholinergic system [17].

The sex-dependent activities of various neurotransmitter systems and brain regions make the different sexes differentially prone to neurological disorders, including Alzheimer’s disease (AD), multiple sclerosis (MS), attention deficit hyperactivity disorder (ADHD), Parkinson’s disease (PD), amyotrophic lateral sclerosis (ALS), and schizophrenia [18]. These brain disorders largely manifest in disturbed social behavior [19] and pose a huge global disease burden [20] affecting millions of people [21]. Certain neuromodulatory drugs, including atypical antipsychotics [22], antidepressants [23], and cholinesterase inhibitors [24], have been used increasingly over the past decade, either alone or in combination [25], to treat these disorders in both sexes. The most common FDA approved drugs are Risperidone (atypical antipsychotic), which primarily blocks dopaminergic D_2_ receptors, as well as serotonergic 5HT_2_A, and α-adrenergic receptors [26]; Paroxetine (antidepressant), which is a selective serotonin reuptake inhibitor, but also shows some norepinephrine reuptake inhibition [27]; and Donepezil, which is a reversible acetylcholinesterase (AChE) inhibitor [23]. Although the therapeutic activities of these drugs against various psychiatric disorders are well-studied, almost 80% of the research involving rodent models in behavioral neuropharmacology and biomedicine is based on male animals only [28]. This situation has improved following certain guidelines by the NIH and other grant awarding bodies, but the data on sex-dependent differences in behavioral neuropharmacology, particularly in social behavior, are still limited [29]. Moreover, how these sex-dependent effects of neuromodulatory drugs on social behavior are modulated by certain environmental factors, like environmental stress, is also not very well studied.

Stress, being a part of everyday life, affects health in multiple ways [30], causing conditions like anxiety and depression [31]. The differential effects of stress on different neurotransmitter systems are controlled by sex-hormones and corticotrophin releasing hormone (CRH) via the hypothalamus-pituitary-adrenal (HPA) and hypothalamus-pituitary-gonadal (HPG) axes [32]. We have shown in our previous studies on rodents that Risperidone and Donepezil affect cognitive functions, especially memory retrieval, in a sexually dimorphic way under normal and stressed conditions [17,33,34,35]. Keeping in view these above-mentioned points, the present study was designed to assess the sex-dependent neuromodulatory effects of Risperidone, Paroxetine, and Donepezil on normal and stress-induced social interaction in rats.

## 2. Materials and Methods

### 2.1. Animal Groups

**Figure 1:**
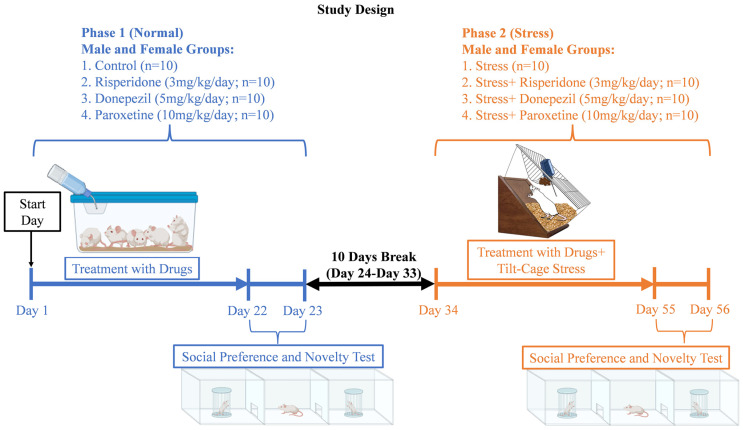
Study Design.

We obtained 40 male and 40 female 8–12 weeks old Wistar rats (130–150 g) from Lab Animal House, ASAB, NUST (H-12 campus), Islamabad. The animals were kept in 16 plastic cages (40 cm × 25 cm × 15 cm), 5 animals in each cage. Standard housing conditions were maintained at 22 ± 2 °C room temperature and 14/10 h light/dark cycle with ad libitum feed and distilled water. In total there were 4 male and 4 female groups, with 10 animals in each group (Figure 1). The 4 male groups included male control group, male Risperidone (3 mg/kg/day) treated group, male Donepezil (5 mg/kg/day) treated group, and male Paroxetine (10 mg/kg/day) treated group. Similarly, the 4 female groups were female control group, female Risperidone (3 mg/kg/day) treated group, female Donepezil (5 mg/kg/day) treated group, and female Paroxetine (10 mg/kg/day) treated group.

### 2.2. Drugs and Doses

The drugs used in this study are as follows: Risperidone, Donepezil, and Paroxetine, which were purchased from a local pharmaceutical company. All other chemicals were obtained from Sigma Aldrich, St. Louis, MO, USA. For the preparation of Donepezil and Paroxetine doses, each drug was directly dissolved in the drinking water of their respective groups. In contrast, Risperidone, being insoluble in water, was first dissolved in 10% glacial acetic acid, and later normalized with NAOH to maintain the pH at 6.5–6.8. After that, this solution was mixed in the drinking water of its respective group. The drugs’ doses were prepared weekly based on the body weights and water intake of the animals.

### 2.3. Scheme of Study

The study duration was 56 days (Figure 1). It was divided into two phases, i.e., phase 1 (21 days of drugs treatment) and phase 2 (21 days of drugs treatment with tilt-cage stress). A gap of 10 days, with normal food and water, was provided between the two phases to ensure the removal of the drugs from the body (Figure 1). The treatment was conducted in such a way that each group received its own specific drugs during both phases (1 and 2).

During phase 1, the social preference and novelty test was performed after 21 days of drug treatment, on days 22–23. After the 10-day break (days 24–33), during phase 2, the same groups received their same respective drugs with chronic mild tilt-cage (45° angle) stress for an additional 21 days (days 34–54). The social preference and novelty test was again performed on days 55–56.

### 2.4. Social Preference and Novelty Test

Social preference and novelty test was performed in a three-chambered box of size 120 cm × 70 cm × 40 cm, following the already described protocol [36] with few modifications. The test rat was placed in the center of a three-chambered social interaction box for 5 min habituation. After the habituation was completed, session 1 was conducted, during which a stranger rat (S1) of the same age, weight, and sex as of the test rat, was introduced in a wired cage in one of the chambers, while the other chamber contained an empty wired cage. The test rat was allowed to explore between the empty cage and the S1 rat for 15 min. At the end of session 1, a 20 min break was provided, and the rat was returned to its home cage. During session 2, the S1 rat was kept the same while another stranger rat (S2) of the same age, weight, and sex as of the test rat was introduced into the empty wired cage. The test rat was then allowed to explore between the S1 rat and S2 rat for 15 min. The apparatus was cleaned with 70% ethanol before performing the test on each rat. All activities were recorded using a camera. The distance traveled in the box was measured by placing a transparent sheet on the video on the laptop’s screen and drawing the boundaries of the box on this sheet. Furthermore, the transparent sheet was subdivided into small boxes of 1 cm size (each), and the number of boxes crossed by the animals was counted. Later, these numbers of boxes crossed were converted to the actual size of the three-chambered instrument, which was larger than the box made on the transparent sheet. Similarly, to make track plots, a transparent sheet was placed on the video on the laptop’s screen and the animal’s movement was drawn with the help of a marker pen. This track was then scanned, and a representative picture from each group is presented.

The parameters assessed in the test are, i. Distance (cm) traveled, ii. Time (s) spent in locomotion, iii. Speed (cm/s) of the animals (by dividing the total distance traveled by the animal to the time spent in locomotion), iv. Time (s) spent in each of the three chambers, v. Time (s) spent in interaction (including sniffing, voluntary touching, and bites to the wired cage), and vi. Discrimination index (%) [37] as:

$$\mathrm{Discrimination}\;Index\left(\%\right)=\frac{Time\;Spent\;Interacting\;with\;S1}{Time\;Spent\;Interacting\;with\;Empty\;Cage\;+\;Time\;Spent\;Interacting\;with\;S1}\times 100$$

$$Discrimination\;Index\left(\%\right)=\frac{Time\;Spent\;Interacting\;with\;S2}{Time\;Spent\;Interacting\;with\;S1\;+\;Time\;Spent\;Interacting\;with\;S2}\times 100$$

### 2.5. Statistical Analyses

The graphs were plotted using GraphPad Prism (version 8.0.1) and data were reported as mean ± SEM. Statistical significance was determined using a one-way ANOVA (Analysis of variance) for single-variable data and two-way ANOVA for two variable data, followed by Tukey’s post hoc test. Data normality was assessed by applying the Shapiro-Wilk test. Data with *p* < 0.05 were considered significant. Pearson correlation (r) was used for checking the correlation between the effects of neuromodulatory drugs on locomotion and social interaction (Supplementary Method).

## 3. Results

### 3.1. Sex-Dependent Effects of Neuromodulatory Drugs on Locomotion in Session 1 (Social Preference Session)

**Figure 2:**
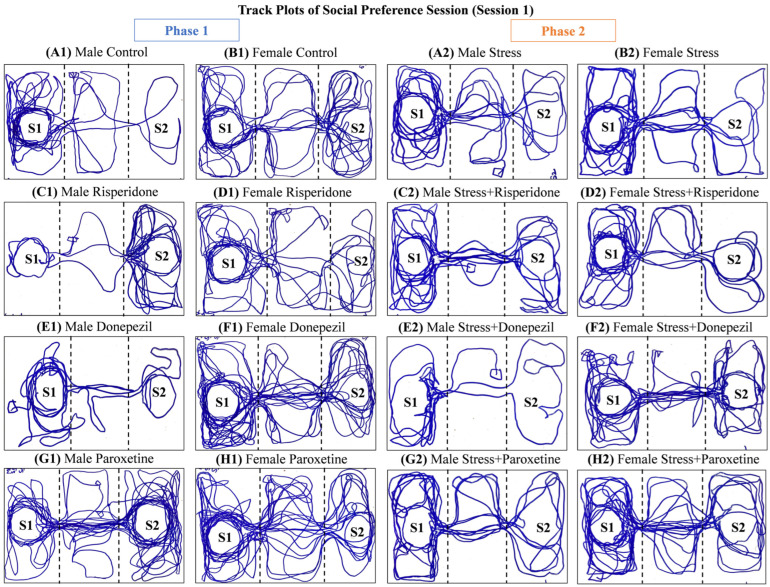
Track plots of One Representative Animal from Each Group during Social Preference Session (Session 1). The plots are of (**Phase 1**) (**A1**) Male Control Group, (**B1**) Female Control Group, (**C1**) Male Risperidone (3 mg/kg/day) Group, (**D1**) Female Risperidone (3 mg/kg/day) Group, (**E1**) Male Donepezil (5 mg/kg/day) Group, (**F1**) Female Donepezil (5 mg/kg/day) Group, (**G1**) Male Paroxetine (10 mg/kg/day) Group, and (**H1**) Female Paroxetine (10 mg/kg/day) Group, and (**Phase 2**) (**A2**) Male Stress Group, (**B2**) Female Stress Group, (**C2**) Male Stress + Risperidone (3 mg/kg/day) Group, (**D2**) Female Stress + Risperidone (3 mg/kg/day) Group, (**E2**) Male Stress + Donepezil (5 mg/kg/day) Group, (**F2**) Female Stress + Donepezil (5 mg/kg/day) Group, (**G2**) Male Stress + Paroxetine (10 mg/kg/day) Group, and (**H2**) Female Stress + Paroxetine (10 mg/kg/day) Group.

**Figure 3:**
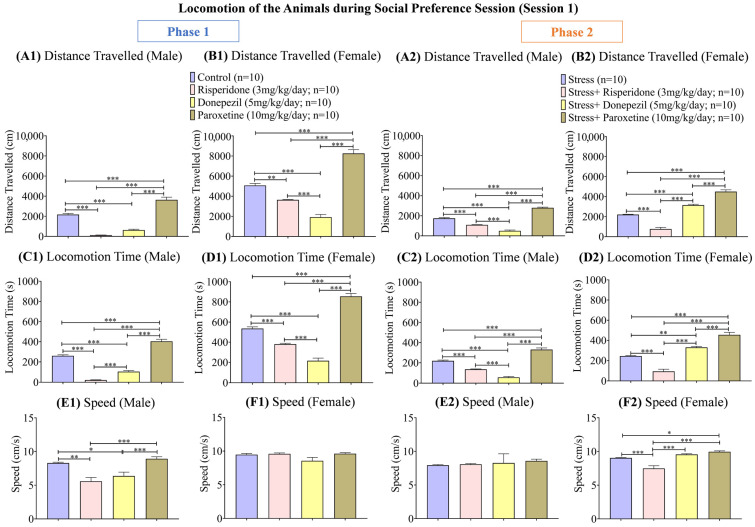
Locomotion of the Animals during Social Preference Session (Session 1). The graphs present a comparison of (**Phase 1**) (**A1**) Distance Traveled (Male), (**B1**) Distance Traveled (Female), (**C1**) Locomotion Time (Male), (**D1**) Locomotion Time (Female), (**E1**) Speed (Male), and (**F1**) Speed (Female) among the Control, Risperidone (3 mg/kg/day), Donepezil (5 mg/kg/day) and Paroxetine (10 mg/kg/day) groups, and (**Phase 2**) (**A2**) Distance Traveled (Male), (**B2**) Distance Traveled (Female), (**C2**) Locomotion Time (Male), (**D2**) Locomotion Time (Female), (**E2**) Speed (Male), and (**F2**) Speed (Female) among the Stress, Stress + Risperidone (3 mg/kg/day), Stress + Donepezil (5 mg/kg/day) and Stress + Paroxetine (10 mg/kg/day) groups. Statistical significance was determined by one-way ANOVA followed by Tukey’s post hoc test. The significant values are denoted as * = *p* < 0.05, ** = *p* < 0.01, *** = *p* < 0.001 while the error bars are represented as mean ± SEM.

Track plots of the animals (Figure 2) and the locomotion graphs (Figure 3) showed differential effects of drugs in both sexes in both phases (1 and 2) of the social preference session.

During phase 1, the distance (cm) traveled was the highest in the male control group (one-way ANOVA; F (3, 36) = 109.9; 2169 ± 114.5; *p* < 0.001) and male Paroxetine (10 mg/kg/day; 3636 ± 269.1; *p* < 0.001) treated group compared to the other two drug-treated male groups (Figure 3(A1)). The trend was similar in female groups, i.e., the control group (one-way ANOVA; F (1.714, 15.43) = 89.98; 5073 ± 208.3; *p* < 0.001) and Paroxetine (10 mg/kg/day; 8256 ± 386.6; *p* < 0.001) treated group showed the highest distance (cm) traveled compared to the other two drug-treated groups (Figure 3(B1)). During phase 2, distance (cm) traveled was reduced in the male stress group (one-way ANOVA; F (1.737, 15.63) = 191.7; 1743 ± 77.52; *p* < 0.001), while it was the highest in male stress + Paroxetine (10 mg/kg/day; 2799 ± 65.60; *p* < 0.001) treated group and the least in male groups treated with Risperidone (3 mg/kg/day; 1097 ± 24.72; *p* < 0.001) and Donepezil (5 mg/kg/day; 503 ± 82.60; *p* < 0.001; Figure 3(A2)). The distance traveled was also reduced in the female stress group (one-way ANOVA; F (2.054, 18.48) = 122.1; 2204 ± 31.98; *p* < 0.001), while it remained the highest in female groups treated with Paroxetine (10 mg/kg/day; 4497 ± 182.6; *p* < 0.001) and Donepezil (5 mg/kg/day; 3149 ± 87.46; *p* < 0.001; Figure 3(B2)).

The locomotion time (s) during phase 1, was the highest in the male control group (one-way ANOVA; F (3, 36) = 175; 260.2 ± 0; *p* < 0.001) and male Paroxetine (10 mg/kg/day; 403.7 ± 0; *p* < 0.001) treated group compared to the other two drug-treated male groups (Figure 3(C1)). The female control group (one-way ANOVA; F (3, 36) = 159.7; 535.2 ± 0; *p* < 0.001) and female Paroxetine (10 mg/kg/day; 855.0 ± 0; *p* < 0.001) treated group also showed the highest locomotion time (s) compared to other two drug-treated female groups (Figure 3(D1)). During phase 2, the male stress + Paroxetine (10 mg/kg/day; 330.2 ± 0; *p* < 0.001) treated group showed the highest, while male stress + Donepezil (5 mg/kg/day; 56.90 ± 0; *p* < 0.001) treated group showed the least locomotion time (s) compared to the male stress group (one-way ANOVA; F (3, 36) = 120.5; 219.0 ± 0; Figure 3(C2)). In contrast, the female stress groups treated with Paroxetine (10 mg/kg/day; 454.7 ± 0; *p* < 0.001) and Donepezil (5 mg/kg/day; 329.9 ± 0; *p* < 0.001) showed the highest locomotion time (s) compared to the female stress group (one-way ANOVA; F (3, 36) = 75.16; 244.1 ± 0; Figure 3(D2)).

The speed (cm/s) during phase 1 was also high in male control (one-Way ANOVA; F (1.347, 12.12) = 11.11; 8.295 ± 0.12; *p* < 0.01) and male Paroxetine (10 mg/kg/day; 8.926 ± 0.29; *p* < 0.001) treated groups compared to the other two drug-treated male groups (Figure 3(E1)), while no significant difference was observed in the speed (cm/s) between any female groups (Figure 3(F1)). During phase 2, there was no significant difference in the speed (cm/s) between any male stress groups (Figure 3(E2)), while among the female groups, the stress + Risperidone (3 mg/kg/day; 7.486 ± 0.40; *p* < 0.001) treated group showed the least speed (cm/s) compared to the stress group (9.039 ± 0.07) and the other two drug-treated groups (Figure 3(F2)).

### 3.2. Sex-Dependent Effects of Neuromodulatory Drugs on Social Preference (Session 1)

**Figure 4:**
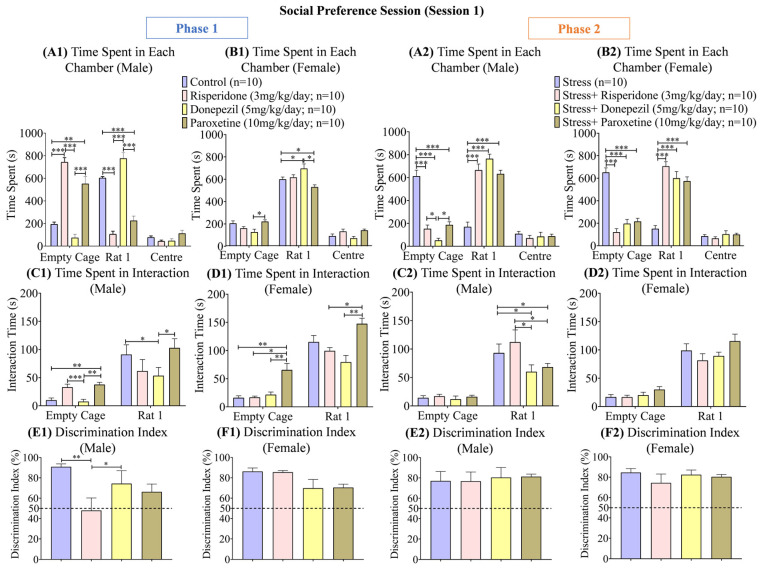
Social Preference Session (Session 1). The graphs present a comparison of **Phase 1** (**A1**) Time Spent in Each Chamber (Male), (**B1**) Time Spent in Each Chamber (Female), (**C1**) Time Spent in Interaction (Male), (**D1**) Time Spent in Interaction (Female), **(E1)** Discrimination Index (Male), and (**F1**) Discrimination Index (Female) among the Control, Risperidone (3 mg/kg/day), Donepezil (5 mg/kg/day) and Paroxetine (10 mg/kg/day) groups, and (**Phase 2**) (**A2**) Time Spent in Each Chamber (Male), (**B2**) Time Spent in Each Chamber (Female), (**C2**) Time Spent in Interaction (Male), (**D2**) Time Spent in Interaction (Female), (**E2**) Discrimination Index (Male), and (**F2**) Discrimination Index (Female) among the Stress, Stress + Risperidone (3 mg/kg/day), Stress + Donepezil (5 mg/kg/day) and Stress + Paroxetine (10 mg/kg/day) groups. Statistical significance was determined by two-way and one-way ANOVAs followed by Tukey’s post hoc test. The significant values are denoted as * = *p* < 0.05, ** = *p* < 0.01, *** = *p* < 0.001 while the error bars are represented as mean ± SEM.

During phase 1, the male control group (two-way ANOVA; F (2, 27) = 201.7; 291.7 ± 159.5; *p* < 0.001) and male Donepezil (5 mg/kg/day; 298.8 ± 238.8; *p* < 0.001) treated group spent a longer time (s) in the S1 chamber than the other two drug-treated male groups, which spent the highest time (s) in the empty cage chamber (Figure 4(A1)). Among the females, the control group (two-way ANOVA; F (2, 27) = 378.2; 298.9 ± 153.9) and all the drug-treated groups spent more time (s) in the S1 chamber (Figure 4(B1)). During phase 2, the male stress group (two-way ANOVA; F (2, 27) = 141.4; 297 ± 158.8; *p* < 0.001) spent less time (s) in the S1 chamber compared to the drug-treated male groups (Figure 4(A2)). The female stress group (two-way ANOVA; F (2, 27) = 165.5; 296.0 ± 178.8; *p* < 0.001) also spent less time (s) in the S1 chamber compared to the drug-treated female groups (Figure 4(B2)).

The interaction time (s) with the S1 rat during phase 1 was the highest in the male control group (two-way ANOVA; F (1, 18) = 74.66; 50.50 ± 40.60; *p* < 0.05) and male Paroxetine (10 mg/kg/day; 70.25 ± 32.55; *p* < 0.05) treated group compared to the other two drug-treated male groups (Figure 4(C1)). Among the female groups, the interaction time (s) with the S1 rat was also the highest in the control group (two-way ANOVA; F (1, 18) = 271.8; 65.70 ± 49.50) and Paroxetine (10 mg/kg/day; 106.6 ± 40.95; *p* < 0.01) treated group compared to the other two drug-treated groups (Figure 4(D1)). During phase 2, the male stress group (two-way ANOVA; F (1, 18) = 80.05; 296.2 ± 184.8; *p* < 0.05) and male stress + Risperidone (3 mg/kg/day; 291.4 ± 162.3; *p* < 0.05) treated group showed the highest interaction time (s) with S1 compared to the other drug-treated male groups (Figure 4(C2)), while no significant difference was observed in the interaction time (s) between any female groups. However, all the female groups spent more time interacting with the S1 rat than the empty cage (Figure 4(D2)).

The discrimination index (%) during phase 1 was the highest in the male control group (one-way ANOVA; F (3, 36) = 6.203; 90.88 ± 2.92; *p* < 0.01) and male Donepezil (5 mg/kg/day; 74.36 ± 12.70; *p* < 0.05) treated group compared to the other two drug-treated male groups (Figure 4(E1)), while no significant difference was observed in the discrimination index (%) between any female groups (Figure 4(F1)). During phase 2, no significant difference was observed between any male groups and any female groups (Figure 4(E2,F2)).

### 3.3. Sex-Dependent Effects of Neuromodulatory Drugs on Locomotion in Session 2 (Social Novelty Session)

**Figure 5:**
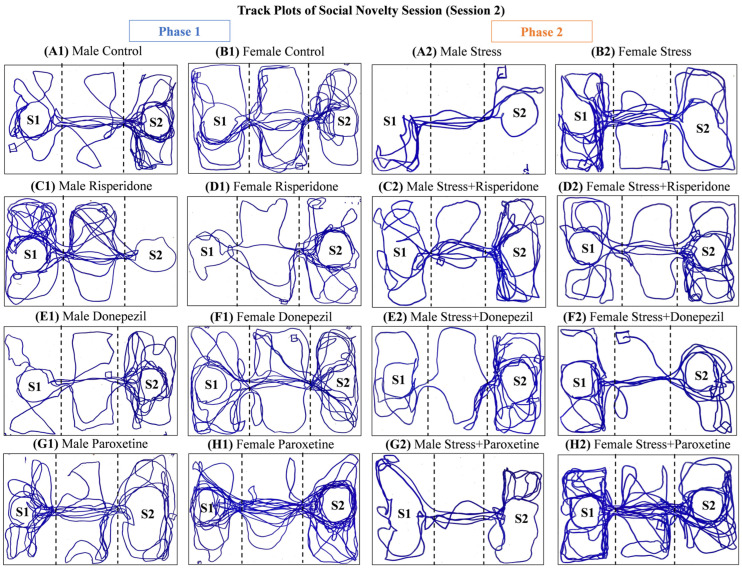
Track plots of One Representative Animal from Each Group during Social Novelty Session (Session 2). The plots are of (**Phase 1**) (**A1**) Male Control Group, (**B1**) Female Control Group, (**C1**) Male Risperidone (3 mg/kg/day) Group, (**D1**) Female Risperidone (3 mg/kg/day) Group, (**E1**) Male Donepezil (5 mg/kg/day) Group, (**F1**) Female Donepezil (5 mg/kg/day) Group, (**G1**) Male Paroxetine (10 mg/kg/day) Group, and (**H1**) Female Paroxetine (10 mg/kg/day) Group, and (**Phase 2**) (**A2**) Male Stress Group, (**B2**) Female Stress Group, (**C2**) Male Stress + Risperidone (3 mg/kg/day) Group, (**D2**) Female Stress + Risperidone (3 mg/kg/day) Group, (**E2**) Male Stress + Donepezil (5 mg/kg/day) Group, (**F2**) Female Stress + Donepezil (5 mg/kg/day) Group, (**G2**) Male Stress + Paroxetine (10 mg/kg/day) Group, and (**H2**) Female Stress + Paroxetine (10 mg/kg/day) Group.

**Figure 6:**
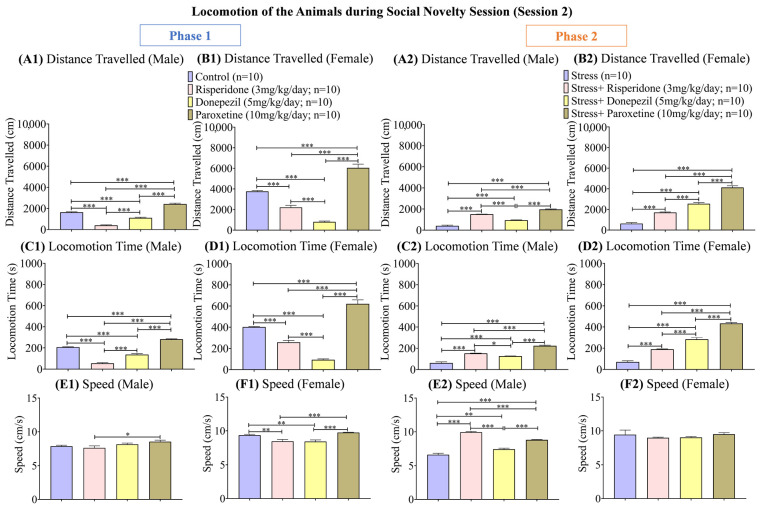
Locomotion of the Animals during Social Novelty Session (Session 2). The graphs present a comparison of (**Phase 1**) (**A1**) Distance Traveled (Male), (**B1**) Distance Traveled (Female), (**C1**) Locomotion Time (Male), (**D1**) Locomotion Time (Female), (**E1**) Speed (Male), and (**F1**) Speed (Female) among the Control, Risperidone (3 mg/kg/day), Donepezil (5 mg/kg/day) and Paroxetine (10 mg/kg/day) groups, and (**Phase 2**) (**A2**) Distance Traveled (Male), (**B2**) Distance Traveled (Female), (**C2**) Locomotion Time (Male), (**D2**) Locomotion Time (Female), (**E2**) Speed (Male), and (**F2**) Speed (Female) among the Stress, Stress + Risperidone (3 mg/kg/day), Stress + Donepezil (5 mg/kg/day) and Stress + Paroxetine (10 mg/kg/day) groups. Statistical significance was determined by one-way ANOVA followed by Tukey’s post hoc test. The significant values are denoted as * = *p* < 0.05, ** = *p* < 0.01, *** = *p* < 0.001 while the error bars are represented as mean ± SEM.

Track plots of the animals (Figure 5) and the locomotion graphs (Figure 6) showed differential effects of drugs in both sexes in both phases (1 and 2) of the social novelty session.

During phase 1, the overall distance (cm) traveled was the highest in the male control group (one-way ANOVA; F (1.945, 17.51) = 168.6; 1639 ± 48.18; *p* < 0.001) and male Paroxetine (10 mg/kg/day; 2420 ± 84.59; *p* < 0.001) treated group compared to the other two drug-treated male groups (Figure 6(A1)). The female control group (one-way ANOVA; F (1.662, 14.95) = 107.7; 3762 ± 91.90; *p* < 0.001) and female Paroxetine (10 mg/kg/day; 6049 ± 357.3; *p* < 0.001) treated group also showed the highest distance (cm) traveled compared to the other two drug-treated female groups (Figure 6(B1)). During phase 2, the male stress group (one-way ANOVA; F (1.169, 10.52) = 222; 174.3 ± 0; *p* < 0.001) showed the least distance (cm) traveled compared to the drug-treated male groups (Figure 6(A2)). This trend was similar in the female stress groups (Figure 6(B2)).

The locomotion time (s) during phase 1 was again the highest in the male control group (one-way ANOVA; F (3, 36) = 236.9; 207.5 ± 0; *p* < 0.001) and male Paroxetine (10 mg/kg/day; 282.0 ± 0; *p* < 0.001) treated group compared to the other two drug-treated male groups (Figure 6(C1)). Among the female groups, locomotion time (s) was the least in Donepezil (5 mg/kg/day; 93.70 ± 0; *p* < 0.001) treated group compared to the control group (one-way ANOVA; F (3, 36) = 112.3; 401.7 ± 0; *p* < 0.001) and the other two drug-treated groups (Figure 6(D1)). During phase 2, the male stressed group (one-way ANOVA; F (3, 36) = 126.5; 61.90 ± 0; *p* < 0.001) followed by male stress + Donepezil (5 mg/kg/day; 126.7 ± 0; *p* < 0.001) treated group showed the least locomotion time (s) compared to the other two drug-treated male groups (Figure 6(C2)). In the female groups, the stress group (one-way ANOVA; F (3, 36) = 204.9; 70.50 ± 0; *p* < 0.001) followed by the stress + Risperidone (3 mg/kg/day; 189.9 ± 0) group showed the least locomotion time (s) compared to the other two drug-treated groups (Figure 6(D2)).

The speed (cm/s) during phase 1 was the highest in the male Paroxetine (10 mg/kg/day; 8.563 ± 0.19; *p* < 0.05) treated group compared to the male control group (one-way ANOVA; F (3, 36) = 3.490; 7.890 ± 0.14) and the other two drug-treated male groups (Figure 6(E1)), while in females, the control group (one-way ANOVA; F (3, 36) = 13.59; 9.354 ± 0.12; *p* < 0.01) and Paroxetine (10 mg/kg/day; 9.745 ± 0.04; *p* < 0.001) treated group showed the highest speed (cm/s) compared to other two drug-treated groups (Figure 6(F1)). During phase 2, the speed (cm/s) was the least in the male stress group (one-way ANOVA; F (3, 36) = 103.2; 6.629 ± 0.21; *p* < 0.001) and male stress + Donepezil (5 mg/kg/day; 7.440 ± 0.15; *p* < 0.001) treated group compared to other two drug-treated male groups (Figure 6(E2)). On the other hand, no female group showed any significant difference in their speed (cm/s; Figure 6(F2)).

### 3.4. Sex-Dependent Effects of Neuromodulatory Drugs on Social Novelty (Session 2)

**Figure 7:**
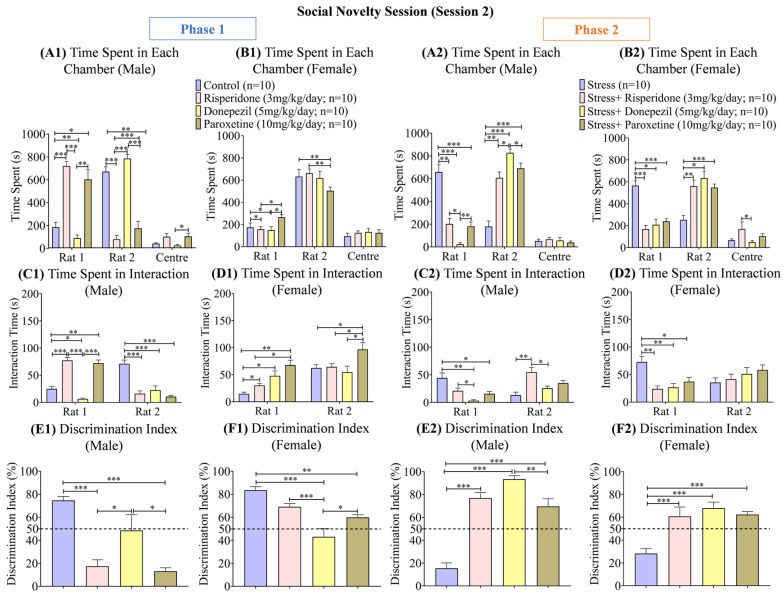
Social Novelty Session (Session 2). The graphs present a comparison of (**Phase 1**) (**A1**) Time Spent in Each Chamber (Male), (**B1**) Time Spent in Each Chamber (Female), (**C1**) Time Spent in Interaction (Male), (**D1**) Time Spent in Interaction (Female), (**E1**) Discrimination Index (Male), and (**F1**) Discrimination Index (Female) among the Control, Risperidone (3 mg/kg/day), Donepezil (5 mg/kg/day) and Paroxetine (10 mg/kg/day) groups, and (**Phase 2**) (**A2**) Time Spent in Each Chamber (Male), (**B2**) Time Spent in Each Chamber (Female), (**C2**) Time Spent in Interaction (Male), (**D2**) Time Spent in Interaction (Female), (**E2**) Discrimination Index (Male), and (**F2**) Discrimination Index (Female) among the Stress, Stress + Risperidone (3 mg/kg/day), Stress + Donepezil (5 mg/kg/day) and Stress + Paroxetine (10 mg/kg/day) groups. Statistical significance was determined by two-way and one-way ANOVAs followed by Tukey’s post hoc test. The significant values are denoted as * = *p* < 0.05, ** = *p* < 0.01, *** = *p* < 0.001 while the error bars are represented as mean ± SEM.

During phase 1, the male control group (two-way ANOVA F (2, 27) = 171.3; 299.1 ± 190.6; *p* < 0.001) and male Donepezil (5 mg/kg/day; 300.0 ± 243.6; *p* < 0.001) treated group spent the highest time (s) in the S2 chamber compared to the other two drug-treated male groups, which spent the highest time (s) in the S1 chamber (Figure 7(A1)). Among the female groups, the control group (two-way ANOVA; F (2, 27) = 158.6; 301.0 ± 167.2; *p* < 0.01) and Risperidone (3 mg/kg/day; 314.9 ± 174.1; *p* < 0.01) treated group spent the highest time (s) in the S2 chamber. The time (s) spent by the female control group (*p* < 0.05) was also high in the S1 chamber, along with the female Paroxetine (10 mg/kg/day; 298.1 ± 110.8; *p* < 0.05) treated group (Figure 7(B1)). During phase 2, the male stress group (two-way ANOVA; F (2, 27) = 154.3; 296.2 ± 184.8; *p* < 0.001) spent the least time (s) in the S2 chamber compared to the drug-treated male groups (Figure 7(A2)). The trend was similar in the female stress groups (Figure 7(B2)).

The interaction time (s) with the S2 rat during phase 1 was the highest in the male control group (two-way ANOVA; F (1, 18) = 14.78; 48.00 ± 23.10; *p* < 0.001), while the male groups treated with Risperidone (3 mg/kg/day; 46.70 ± 30.60; *p* < 0.001) and Paroxetine (10 mg/kg/day; 41.40 ± 30.70; *p* < 0.001) showed higher interaction with the S1 rat (Figure 7(C1)). Among the female groups, the control group (two-way ANOVA; F (1, 18) = 28.21; 38.50 ± 23.90; *p* < 0.01) spent the least time (s) interacting with the S1 rat, while the drugs treated groups showed no prominent difference in interaction time (s) with the S1 rat and S2 rat (Figure 7(D1)). During phase 2, the male stress group (two-way ANOVA; F (1, 18) = 9.717; 28.70 ± 15.50; *p* < 0.05) showed the highest interaction time (s) with the S1 rat, while the male groups treated with Risperidone (3 mg/kg/day; 38.00 ± 17.00; *p* < 0.05) and Paroxetine (10 mg/kg/day; 25.30 ± 9.70) showed a higher time (s) in interaction with the S2 rat (Figure 7(C2)). Of the female groups, the stress group (two-way ANOVA; F (1, 18) = 0.6800; 54.05 ± 18.55; *p* < 0.001) showed the highest interaction time (s) with the S1 rat, while the drug-treated groups again showed no prominent difference between interaction time (s) with the S1 rat and S2 rat (Figure 7(D2)).

The discrimination index (%) was the highest in the male control group (one-way ANOVA; F (3, 36) = 21.45; 74.61 ± 3.43; *p* < 0.001) and male Donepezil (5 mg/kg/day; 48.68 ± 13.55; *p* < 0.05) treated group compared to the other two drug-treated male groups (Figure 7(E1)). In females, the discrimination index (%) was the least in the Donepezil (5 mg/kg/day; 43.17 ± 7.22; *p* < 0.001) treated group compared to the control group (one-way ANOVA; F (3, 36) = 11.44; 83.59 ± 3.09; *p* < 0.001) and the other two drug-treated female groups (Figure 7(F1)). During phase 2, the discrimination index (%) was the least in the male stress group (one-way ANOVA; F (3, 36) = 24.84; 15.42 ± 4.79; *p* < 0.001), while it was highest in the male stress + Donepezil (5 mg/kg/day; 93.43 ± 3.08; *p* < 0.001) treated group compared to the other two drug-treated male groups (Figure 7(E2)). The female stress group (one-way ANOVA; F (3, 36) = 12.75; 28.32 ± 4.27; *p* < 0.001) also showed the lowest discrimination index (%) compared to the three drug-treated female groups (Figure 7(F2)). Correlation graphs were also made to assess the relationship between the effect of drugs on locomotion and social interaction, and no direct correlation was found (Supplementary Figure S1).

## 4. Discussion

Sexual dimorphism is exhibited by various species in their behavioral dimensions, such as communication style, aggression, affiliation, and response to stress [38,39]. It plays important role in the diagnosis, management, and treatment of various neurological disorders in humans [40]. The sex-dependent behavioral differences are due to the differential activities of various brain regions and their associated neurotransmitter systems [38,39], which can also be affected by environmental stress. For example, stress is reported to increase dopamine and serotonin concentrations in subcortical regions controlling social behavior in females, while increasing the metabolites of these neurotransmitters in males [15,41]. Similarly, the expression of mAChRs in the ventral hippocampus is reported to increase due to stress in male mice only [42]. Such sex-dependent differences in brain regions and their associated neurotransmitter systems suggest differential effects of certain neuromodulatory drugs on these systems, which need to be studied to produce better targeted interventions for neurological disorders.

The social preference and novelty test was performed in the current study to test the social interaction and locomotion of the animals. Locomotion was increased after Paroxetine treatment in both sexes, particularly in the females, compared to their respective control groups, during both sessions of the test under normal conditions (without stress). This could be attributed to increased extracellular serotonin levels causing hyperactivity in the animals [43], with a reportedly higher response to SSRIs in females only [44], which requires further studies. In contrast, Donepezil treatment caused reduced locomotion in both sexes, as reported previously [45]. However, the differential, sex-dependent effects of the drug on locomotion during both sessions can be attributed to the animals’ social preferences and the effects of sex-hormones on Donepezil-induced disturbance in cholinergic to adrenergic ratio [45], but further studies should be conducted. Risperidone treatment led to an overall reduced locomotion in both sessions of the test in the male groups only, which is likely to be due to the sex-dependent modulation in dopaminergic nigrostriatal and mesolimbic pathways [46,47]. Overall, the effects of Risperidone and Donepezil were more prominent on male groups, while Paroxetine affected both sexes in an almost equal manner.

After stress, the locomotion of male and female stress groups was reduced, possibly due to chronic stress-induced depression and the release of stress hormones [48,49], while Paroxetine treatment again led to an increased locomotion in both sexes, particularly in females. An increased cholinergic to adrenergic ratio, likely due to Donepezil, causes depression, while a reduced ratio causes mania [45]. In the present study, the locomotion of the male Donepezil treated group was again low in phase 2, while the locomotion of the female Donepezil group was increased to a greater extent, possibly due to estrogen-led compensation for the cholinergic to adrenergic ratio [50]. Treatment with Risperidone increased locomotion in males only, while making it worse in females, again suggesting sex-dependent receptor targets in the cortical areas [46,47], which need further exploration. The locomotion of the male and female stress groups was reduced in an equal manner. However, the modulatory effects of Risperidone were more prominent on the locomotion of males, while Donepezil and Paroxetine showed mixed effects on both sexes.

The social interaction assessment of the animals was based on social preference (animal over empty cage) and social novelty (novel animal over familiar animal). The social preference and social novelty were high in the control groups in both sexes, with a discrimination index above 50%, which is usually considered to be a good measure of the animals’ discrimination ability [51]. Paroxetine treatment also led to increased social preference in both sexes under normal conditions (without stress in this case), while it reduced social novelty, leading to a discrimination index below 50%, in males only, probably by specifically blocking neuronal serotonin transporter (SERT), which affects males more and increases their chances of ADHD [52,53]. Donepezil treatment also led to increased social preference but a reduced and equal interaction with both stranger rats during session 2, with a discrimination index of 50%. The reduced locomotion but intact social recognition, particularly in females, indicates that AChE inhibition by Donepezil produces differential effects based on the target area and sex-hormones [45]. Risperidone treatment led to reduced social preference and social novelty, with a discrimination index below 50% in male groups only. This could be due to the specific blockage of D_2_, 5HT_2_A, and/or α-adrenergic receptors by the drug, reportedly higher in males’ NAc, PFC, and amygdala [46,47], the regions in the mesolimbic dopaminergic pathway controlling social behavior. Overall, all three drugs showed more prominent effects on the social interaction of male groups.

After stress, overall social interaction was reduced in all male and female groups. Male and female stress groups showed a high social preference but a reduced social novelty and an associated discrimination index of below 50%. This is usually a coping mechanism for stress where the animal tries to form a social connection, when alone, with a single conspecific, but prefers to stay with a familiar animal in the presence of more than one conspecific [54,55,56,57]. Paroxetine treatment increased the social preference in females only, while the social novelty did not increase much in both sexes. The discrimination index was above 50% in both sexes. This could be attributed to an increased SERT desensitization and reduced emotionality after prolonged Paroxetine exposure, as reported earlier [58]. The Donepezil treated male and female groups, particularly the female group, indicated social preference and social novelty just like under normal conditions, but with a high discrimination index suggesting a prophylactic effect of the drug against chronic stress-induced depression [45]. Meanwhile, the Risperidone treatment increased social preference, social novelty, and discrimination index in male groups, again confirming the drug’s sex-dependent specificity in neuronal targeting [46,47]. The social interaction of both sexes was affected by stress in an equal manner; however, the effects of drugs on different male and female stress groups were complex.

The present study has provided a significant insight into the sex-dependent effects of Risperidone, Donepezil, and Paroxetine in modulating social behavior under normal and stressed conditions. These results can be extrapolated to humans to an extent due to their close behavioral and genetic resemblance to rodents [59]. This study has depicted a real-life scenario in which people use these neuromodulatory drugs under different environmental conditions irrespective of their biological or hormonal states. However, there are some limitations associated with the study, such as that no behavioral indicators of stress were measured. These indicators may help to show behavioral demonstrations of levels of stress at the end of the study duration. Moreover, sex-hormone assessment was not measured during or at the end of the study. Knowing the levels of hormones and their correlation with behavior and drug treatment can provide better insights in future [60]. Additionally, the within-animal design, where animals served as their own controls, provided a robust approach to assessing the specific effects of the drugs in the same animals under normal and stressed conditions. A 10-day break between the two phases to minimize the potential carryover effects of the drugs was chosen in our study. In future, longer duration of no drug time (break time) can be employed to find out if there would be any difference in results. Furthermore, research involving synaptosomal concentrations, receptor expression, and neuronal activities using functional techniques should also be conducted in both rodents and humans.

## 5. Conclusions

The study has shown that different commonly used pharmacological drugs under normal and stressed conditions can affect social behavior in a sex-dependent manner. Results have shown that Risperidone predominately affects the locomotion and social behavior of males, while the effects of Paroxetine and Donepezil vary in both sexes under normal and stressed conditions. Further studies are needed to understand the underlying mechanisms and to develop sex-specific interventions for the treatment of social deficits and mood disorders in the context of broad social dynamics.

## Data Availability

All the data related to this research are contained within the paper.

## Supplementary Materials

The following supporting information can be downloaded at: https://www.mdpi.com/article/10.3390/brainsci13101378/s1. Figure S1: Correlation Between Locomotion Time (s) and Social Interaction Time (s).

## Abbreviations

AChE	Acetylcholinesterase
ADHD	Attention Deficit Hyperactivity Disorder
AD	Alzheimer’s Disease
ALS	Amyotrophic Lateral Sclerosis
ANOVA	Analysis of Variance
ASD	Autism Spectrum Disorder
D_2_	Dopaminergic Receptor 2
FDA	USA Food and Drug Administration
HPA	Hypothalamus-Pituitary-Adrenal
HPG	Hypothalamus-Pituitary-Gonadal
IRB	Internal Review Board
LC	Locus Coeruleus
MS	Multiple Sclerosis
NAc	Nucleus Accumbens
NAOH	Sodium hydroxide
NIH	National Institute of Health
PD	Parkinson’s Disease
S1	Stranger Animal 1
S2	Stranger Animal 2
SEM	Standard Error Mean
SERT	Serotonin Transporter
5HT_1_A	5-Hydroxy tryptamine 1 Receptor A
5HT_2_A	5-Hydroxy tryptamine 2 Receptor A
