# Supplementary material for: Sexually Dimorphic Effects of Neuromodulatory Drugs on Normal and Stress-Induced Social Interaction in Rats
Source: Brain Sci. 2023 Sep 27;13(10):1378. doi: 10.3390/brainsci13101378 (PMC10604924; doi:10.3390/brainsci13101378)
Supplement: Supplementary file 1 [file brainsci-13-01378-s001.zip › brainsci-2604675-supplementary.pdf]

Sexually Dimorphic Effects of Neuromodulatory Drugs on Normal and Stress-Induced Social Interaction in Rats

Sara Ishaq and Touqeer Ahmed *

Neurobiology Laboratory, Department of Healthcare Biotechnology, Atta-ur-Rahman School of Applied
Biosciences, National University of Sciences and Technology, Islamabad 44000, Pakistan;
sarahishaq1622@gmail.com
* Correspondence: touqeer.aahmed@gmail.com or touqeer.ahmed@asab.nust.edu.pk

Supplementary Method

Assessment of Correlation Between Locomotion Time (s) and Social Interaction Time (s)

A Pearson (r) correlation was assessed between the locomotion time (s) and the social interaction time (s) of the groups to check whether the effect of the drugs and stress on locomotion influences their effect on social interaction. Graphs were made with locomotion time (s) on left y-axis and social interaction time (s) on right y-axis with animal groups on x-axis for each session (session 1 and 2) both as normal and after stress for all the male and female groups.

Results

From the correlation graphs, only significant ($p<0.05$) correlation between locomotion time (s) and social interaction time (s) was found in the female groups during session 1 of phase 1 (normal) of the social preference and novelty test (Supplementary Figure B1). While no correlation was found in both sessions (1 & 2) during both phases (1 & 2) of the test among the male groups. Female groups also did not show any correlation during phase 2 (after stress) of the session 1 and during both phases (1 & 2) of session 2 of the test. The individual Pearson correlation (r) and p values for both sessions (1 & 2) during both phases (1 & 2) of the test are shown (Supplementary Figure A1-D2). Although the effects of drugs were prominent on the locomotion of different male and female groups as well as their social interaction under normal and stressed conditions, but it was found that these effects of drugs on locomotion did not affect the animals' tendencies to

interact socially with the novel animals. This means that the effects of drugs on locomotion and on social interaction, both under normal and stressed conditions, are two independent variables and have no direct relation with each other.

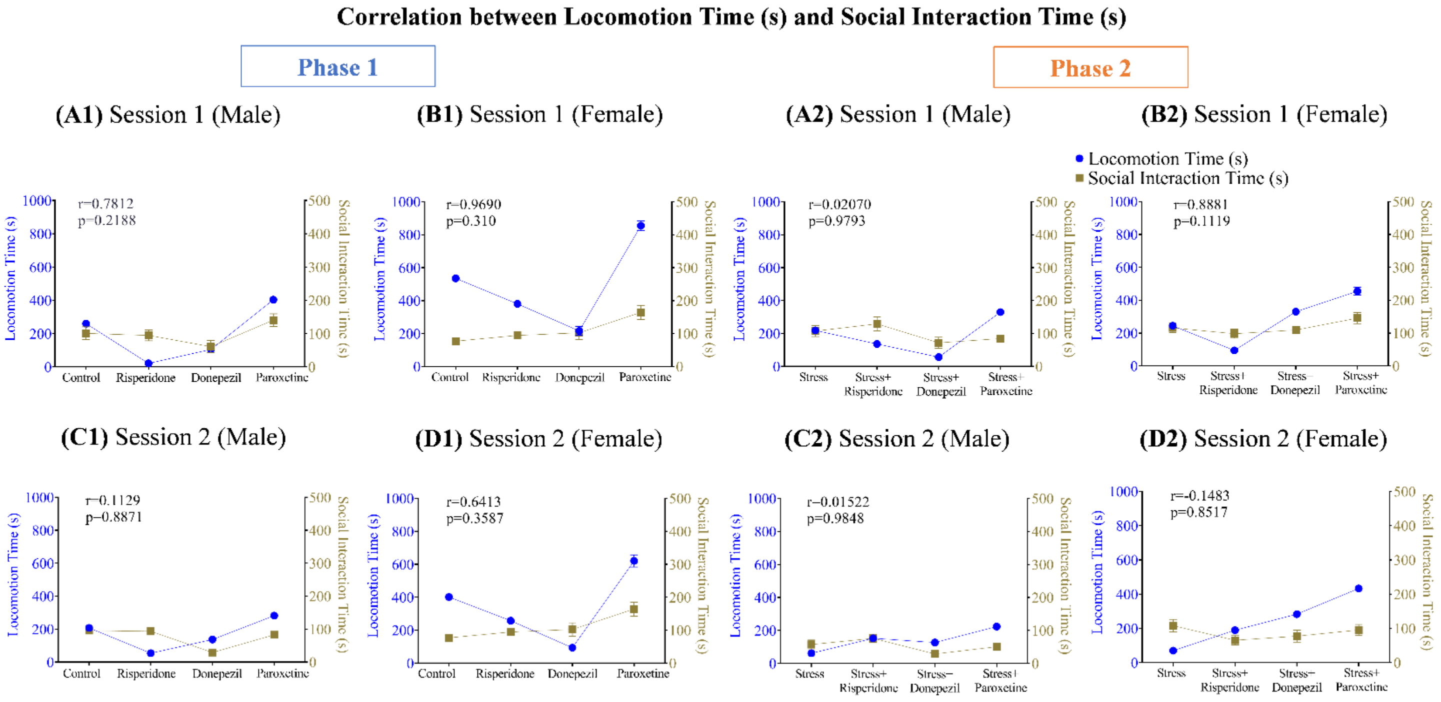

Supplementary Figure S1. Correlation Between Locomotion Time (s) and Social Interaction Time (s). The graphs represent correlation in **Phase 1 (A1)** Session 1 (Male), **(B1)** Session 1 (Female), **(C1)** Session 2 (Male) and **(D1)** Session 2 (Female) among the Control, Risperidone (3mg/kg/day), Donepezil (5mg/kg/day) and Paroxetine (10mg/kg/day) groups, and **Phase 2 (A2)** Session 1 (Male), **(B2)** Session 1 (Female), **(C2)** Session 2 (Male), and **(D2)** Session 2 (Female) among the Stress, Stress+Risperidone (3mg/kg/day), Stress+Donepezil (5mg/kg/day) and Stress+Paroxetine (10mg/kg/day) groups. Error bars are represented as mean±SEM. r=Pearson correlation coefficient, p=correlation significance.
